# An Adaptive CBPR Approach to Create Weight Management Materials for a School-Based Health Center Intervention

**DOI:** 10.1155/2013/978482

**Published:** 2013-07-29

**Authors:** Andrew L. Sussman, Carolyn Montoya, Olaf Werder, Sally Davis, Nina Wallerstein, Alberta S. Kong

**Affiliations:** ^1^Department of Family and Community Medicine, University of New Mexico, Albuquerque, NM 87131-0001, USA; ^2^College of Nursing, University of New Mexico, Albuquerque, NM 87131-0001, USA; ^3^Department of Media and Communications, The University of Sydney, Sydney, NSW 2006, Australia; ^4^Department of Pediatrics, University of New Mexico, Albuquerque, NM 87131-0001, USA

## Abstract

*Purpose*. From our previous clinical work with overweight/obese youth, we identified the need for research to create an effective weight management intervention to address the growing prevalence of adolescent metabolic syndrome. Formative assessment through an adaptive community-based participatory research (CBPR) approach was conducted toward the development of a nutritional and physical activity (DVD) and clinician toolkit for a school-based health center (SBHC) weight management intervention. *Methods*. We first conducted parent and adolescent interviews on views and experiences about obesity while convening a community advisory council (CAC) recruited from two participating urban New Mexico high schools. Thematic findings from the interviews were analyzed with the CAC to develop culturally and developmentally appropriate intervention materials. *Results*. Themes from the parent and adolescent interviews included general barriers/challenges, factors influencing motivation, and change facilitators. The CAC and university-based research team reached consensus on the final content of nutrition and physical activity topics to produce a DVD and clinician toolkit through six monthly sessions. These materials used in the SBHC intervention resulted in a greater reduction of body mass index when compared to adolescents receiving standard care. *Conclusions*. Formative assessment using an adaptive CBPR approach resulted in the creation of culturally and age appropriate weight reduction materials that were acceptable to study participants. This trial is registered with ClinicalTrials.gov NCT00841334.

## 1. Introduction

An unfortunate consequence of the childhood and adolescent obesity epidemic is the emergence of metabolic syndrome, a condition that was historically seen in adults. Thirty percent of obese adolescents in the United States meet criteria for metabolic syndrome [1] and the prevalence is increasing [2]. Consistent with adult rates, prevalence of metabolic syndrome in adolescents is highest among Hispanics, followed by African Americans when compared to non-Hispanic whites [3], while type 2 diabetes is increasing among American Indian adolescents more than any other racial/ethnic group in the United States [4]. 

Weight loss through behavioral modification is an appropriate first step in the primary care treatment of metabolic syndrome [5]. Nevertheless, almost 80% of pediatricians report frustration with their ability to make an impact on obesity [6] and many providers feel they do not have the tools to effectively address lifestyle modification for weight loss [7]. Schools, where children and adolescents spend the majority of their time, are promising venues for intervention work [8]. A recently released Institute of Medicine report strongly endorsed this perspective, citing schools as a “national focal point” in efforts to address obesity in children and adolescents [9]. As several systematic reviews demonstrate, however, there is a paucity of obesity intervention research in high school settings targeting older adolescents and even less in school-based health centers (SBHCs) [10–12]. Located in the school setting, SBHCs offer a unique opportunity for health care delivery. SBHCs have several compelling features for a successful lifestyle intervention program to treat overweight/obesity and prevent development of metabolic syndrome: (1) SBHC clinicians have more access to adolescents than community health care providers; (2) adolescents have better compliance and followup in school-based clinics (both of which are needed for weight loss and maintenance); and (3) SBHCs focus on early identification of high-risk problems [13–18].

In response to the obesity epidemic and adolescents' interest in promoting physical activity and healthy eating, our research was built on a long-standing partnership between the University of New Mexico School-based Health Center Program and an urban school community. Through previous engagement with school-based clinicians and adolescent students, we reached consensus on collective interest to create a DVD to help adolescents achieve weight loss and a clinician tool kit to assist providers in their motivational interviewing efforts [19]. This partnership led to the creation of a culturally and developmentally appropriate overweight/obesity intervention program, Adolescents Committed to Improvement of Nutrition and Physical Activity (ACTION). Consistent with American Academy of Pediatric Expert Committee recommendations regarding the treatment of adolescent overweight/obesity, the ACTION intervention featured eight sessions with a health care provider using motivational interviewing to encourage behavior change toward active living and healthful eating [20]. This paper describes the use of an adaptive community based participatory research (CBPR) process through which formative research data were collected for development of an SBHC intervention for prevention of metabolic syndrome in multiethnic high school students. 

## 2. Methods

### 2.1. Overall Design: Formative Assessment Using a CBPR Approach

Formative research is used to understand people's beliefs, perceptions, and behaviors for the purpose of developing culturally appropriate behavioral change programs [21]. Health researchers typically include a formative “stage” of research in their study designs to ensure that the identification of such cultural attributes and contextual factors can be integrated into intervention activities [22–24]. Given our interest in developing intervention tools (DVD and Toolkit) to help school-based providers and multiethnic adolescents, we designed this study to ground formative assessment through a CBPR partnership [25, 26]. Our review of the literature revealed that use of CBPR in formative research is quite limited and we have not found examples of other manuscripts reporting this type of approach in the context of an SBHC study [27]. We provide further detail showing the link between CBPR principles and our research steps in Table 1. 

To maximize time for development and completion of the DVD and toolkit in one year, recruitment for semistructured interviews with overweight/obese adolescents and parents of overweight/obese adolescents was concurrent with recruitment of overweight/obese adolescents and their parents to the Community Advisory Council (CAC). Adolescents and parents were recruited from the two participating urban high schools with long-standing school-based health centers. Interviews with participants unknown to the CAC were conducted to obtain fuller disclosure of sensitive information about living with obesity that the University research team felt may have been too sensitive to discuss openly among the CAC members. In the second year of the study, the intervention was implemented using these materials. The study protocol was approved by the University of New Mexico Human Research Protections Office and the Urban Schools' Research, Development, and Accountability Department. 

### 2.2. Student and Parent Interviews

#### 2.2.1. Participant Recruitment

A purposeful sampling strategy that reflected the culturally diverse school population was used to recruit overweight/obese adolescent and parent dyads. Given the intent to understand perceptions of obesity and barriers/facilitators to weight loss, we focused recruitment efforts on overweight/obese adolescents. We also conducted “extreme case” sampling to identify adolescents who self-reported success in achieving weight loss (Table 2). We believed that these more unusual cases would lead to insights relevant to our intervention development. All of the adolescents were referred to the research team by participating school-based health center providers and partnering school staff (e.g., counselors, school nurse, and teachers). We interviewed parents most responsible for food preparation in the household. The research team coordinated recruitment efforts with collaborators at the participating high schools including the Activity Directors, School Health Advisory Council members, Student Senate members, and SBHC clinicians. 

#### 2.2.2. Data Collection

Adolescent and parent interview guides were designed to assess concordance of perspectives in the following areas: media use and information seeking strategies, definitions of health, health concerns (weight related), strategies/approaches to weight loss, barriers/facilitators to health in the school and home environment, and suggested content and style for the DVD. The interview guide was designed to be neutral to potentially stigmatizing perspectives about adolescent obesity and contributing factors. Adolescents and parents were interviewed separately and interviews were conducted at times and locations convenient for the family, mostly in their homes and some at the University. Consent/assent forms were mailed in advance for review and respondents were formally consented at the start of each interview. Most interviews lasted approximately one hour and each respondent received a $20 reimbursement for their time.

#### 2.2.3. Analytic Process

Following an iterative analytic process, the multidisciplinary research team—including a medical anthropologist, adolescent medicine physician, primary care nurse practitioner, health communications researcher, and community based participatory research practitioner—reviewed sets of 3 to 4 transcripts independently, identifying key themes relevant to the subsequent creation of the intervention materials. The team reviewed emergent themes and developed a preliminary analytic framework. We specifically focused on responses between adolescents and parents, seeking both complementary and divergent perspectives on key elements for the DVD and provider toolkit materials including content, messaging, and context of use. Ongoing data collection and analysis continued until 15 interviews had been conducted (7 students, 8 parents; the sample consisted of 6 parent-child pairs), at which point the research team reached consensus that the full range of interpretive themes had been identified. At that point, the medical anthropologist (ALS) coded each interview in the qualitative data analysis software program NVivo 8 to facilitate text retrieval and create summary reports [28]. Further demographic details of the student and parent interviewees are presented in Table 2. 

### 2.3. Acceptability and Satisfaction with ACTION Components

Following the intervention and as part of a broader process evaluation effort designed to examine a range of implementation issues, we assessed adolescent and parent views of acceptability and satisfaction with the materials developed in the formative phase. 

#### 2.3.1. Data Collection

Intervention students were each asked to fill out questionnaires with Likert scale ratings of the ACTION DVD, toolkit handouts, and overall satisfaction with the intervention. Parent questionnaires focused on materials they received as part of the ACTION intervention, including a parent newsletter as well as views of the toolkit handouts and overall satisfaction with their child's participation in ACTION. 

#### 2.3.2. Data Analysis

Descriptive summaries of the questionnaires for each group were assessed. Frequencies, confidence intervals, and summary statistics were calculated for all variables.

### 2.4. CBPR Process: Formation of the Community Advisory Council

Once the interviews and a preliminary analytic summary had been developed, we convened the Community Advisory Council (CAC) as a forum in which to translate themes from the interviews into specific strategies and materials to be used in the intervention. Our goal was to form a group with similar demographic characteristics to the targeted study population: Hispanic, African American, and American Indian overweight/obese students and their parents. Given our expectation of attrition, we recruited a total of 16 participants (divided equally between students and parents, including four parent-child pairs). 

We began a series of six consecutive monthly meetings from December 2008 to May 2009 at the University of New Mexico during which the DVD and toolkit were developed. The CAC met on a less frequent basis throughout the second year of the study (August 2009 to May 2010) to provide implementation guidance and feedback. Meetings were held in the evenings to accommodate work and school schedules. We provided a full dinner, free parking, and reimbursement for travel expenses ($10) for all participants immediately following each meeting. CAC members were also informed that all would receive the DVD resulting from their efforts as well as a personal DVD player. 

Sessions were typically comoderated by the DVD producer and study coinvestigator (ALS). However, when meetings were focused on topics of nutrition and/or physical activity, sessions were led by the research team registered dietician and fitness expert. This approach addressed a central CBPR principle in fostering information exchange between community members and the research team. The meetings were designed to be cyclical and iterative as a way to reach consensus and tailor evidence-based strategies to ensure the cultural and age appropriateness of the DVD and toolkit materials as well as stylistic elements (e.g., music and images). Given the flexible, participatory nature of these meetings, CAC members shaped the process by volunteering to test potential data collection methods (e.g., maintaining a food diary) and requesting further information of the research team to facilitate colearning (connections between obesity and health). 

## 3. Results

*Overview. *We have organized the presentation of results to reflect the sequential process of data collection leading to the creation of the ACTION intervention materials. We first present the thematic findings from key informant interviews that provided guidance to the research team and the CAC. We then describe how the CAC translated this input into the DVD and provider toolkit. Lastly, we include process evaluation measures reflecting adolescent and parent views of these materials following the ACTION intervention. 

*(I) Key Interview Themes*

*(1) Media Use.* Adolescents consistently indicated use of the internet as a primary source for both entertainment and information seeking. Most students reported easy access to the internet either at home and/or during school. As one adolescent female indicated

*“A lot of people get on the internet. A lot of teenagers I know use the internet and look up information that way. If it's something that looks interesting they'll go look at it.”*

Further, most adolescents described using the internet as a way to gather health information about a range of issues, including diet and exercise. They viewed the internet as a reliable source of information that could be accessed in private. Another female adolescent described using the internet for health information and as a basis for discussion with her doctor:

*“They have that pyramid, I'm not sure what it's called. And you can look at how many calories you're supposed to take in a day and I looked at those and I just started doing that last month with my doctor.”*

*(2) “Functional” Definition of Health.* In each of the interviews, we asked adolescents how they define health and what that means to them. In some cases, they identified the association between being overweight/obese and the future development of health problems such as diabetes and heart disease. Several of these teens were aware of these risks given family members dealing with these conditions. However, the most striking theme to result from these interviews related to how being overweight/obese limited adolescents activities in the present. Overall, they were less concerned with the long-term consequences and referred to a “functional” definition of health that focused on the degree to which their weight impacted the ability to engage in activities of interest. One male adolescent, echoing the response of several others, responded to the question “what does it mean to be healthy?” by stating “Being healthy to me is being active.” 

*(3) Barriers to Weight Loss in the School.* Not surprisingly, both teens and parents reported a wide range of challenges related to their struggles with food choices and physical activity in their weight control efforts. Teens consistently noted that healthful foods/drinks were not available in the school setting. Adolescents regularly purchased foods from vendor carts but were frustrated by the lack of healthful options such as water rather than soda. In addition to expressing a need for healthier snacks from vendors, the teens also pointed to unhealthful meal choices at school. As a potential way to address this problem, one teen stated

*“…mostly not sell as much pizza, ‘cause like everywhere you go they sell pizza and burritos, like breakfast burritos and sometimes even regular burritos at lunch. Even if they change the menu up a little bit that'd be better because it wouldn't be the same thing every day—just burritos, burritos or pizza, pizza, pizza.”*

Parents consistently agreed with adolescents about the need for schools to offer foods that are both appealing and offer higher nutritional value. 

Another challenge identified by both the teens and the parents is the lack of opportunity for exercise in the school setting. Teens emphasized the need for more physical activities not related to formal participation on sports teams. Parents focused their concern on curricular issues such as the need to require a gym class for all four years of High School rather than just one semester. 

*(4) Strategies to Achieve Weight Loss*. Several adolescents interviewed reported success in their efforts to lose weight. Although a range of strategies were described—mostly involving diets and increases in physical activity—adolescents expressed the importance of internal motivation as the essential element in weight loss initiation efforts. Consistent with the “functional” definition described above, students identified personal goals such as making a sports team or improving their appearance for a significant school event such as the prom as a reason to make physical activity and nutritional changes. Similarly, there was consensus among the parents that it was their obligation to serve as a principal source of motivation. As one parent said

*“I would have to say it (motivation) would have to be left on the parent's shoulders. I think if they see the parents motivated to want to exercise and take care of their body and eat healthy then the children are going to see that and that's going to help them. ‘Okay, mom's doing this, maybe we should do this, too.'”*

*(5) Parent Views on Changing Home Environment.* While we mostly focused these interviews on the school environment, we specifically engaged parents about the challenges they confront in weight loss efforts at home. The goal of these discussions was to elicit strategies that could be included in the home-based component of the ACTION intervention. Parents echoed the adolescents in recognizing the broad importance of motivation as a way to initiate health behavior changes. As one mother indicated

* “Some ideas on how to motivate your kids…ideas on how to keep them wanting to live a good lifestyle, you know, healthy lifestyle and full of exercise. That's a real big one for me, motivation. And then just trying to change their mentality, change their way of thinking so that it's stuck in their head. My goodness, it's hard.”*

We also asked parents for suggestions of informational needs about nutrition and/or physical activity that would be useful to them at home. Another mother described interest in having a list of practical comparisons of food choices:

*“I always find it interesting…when they do comparisons like which is actually the healthier (food) and you're going, ‘I don't know, they're actually both kind of bad, but which is actually the better choice?'”*

*(6) Input on DVD Content.* Lastly, the interviews provided a rich opportunity to gather input on content and stylistic elements of the proposed DVD. Consistent with the theme of internal motivation, most adolescents requested that the DVD content did not scare or threaten by presenting adverse health consequences of overweight/obesity. Instead, they emphasized the importance of featuring teens in the DVD that were currently overweight/obese so that adolescents would relate more to the content:

*“Include overweight people…and show that they're really working hard and then show them successfully doing it and get in a normal (weight) range.”*

Reflecting common elements of teen culture, most of the teens suggested using popular activities as a vehicle through which the messages could be promoted. This included differing forms of dance, kickboxing, use of appropriate music, and presenting these activities with careful attention to style through rapid cutting to different camera angles and shots. When asked what should be in the DVD, one male adolescent quickly responded

*“Dancing, I'm pretty sure ‘cause everyone loves to dance; say if you want to learn how to fight and get in shape and defend yourself there's kickboxing, so pretty much those things.”*

*(II) Translating Themes to DVD and Toolkit with the Community Advisory Council*. As the research team finalized the analysis of the parent and adolescent interviews, we convened the CAC and held the first of six consecutive monthly sessions. The first session was primarily an orientation to the project and subsequent meetings were led by content expert moderators from the research team to identify and refine intervention content. Below, we provide a list of the major themes and the resulting strategies that were integrated into the DVD and toolkit. Table 3 provides a summary of these themes and strategies. 

*(1) Media Use and Information Seeking*. Interviewed adolescents expressed reliance on using the internet for a range of informational needs. CAC participants confirmed this and agreed with a proposal to include websites in the intervention materials for adolescents to access nutritional and physical activity information. During one of the sessions, we reviewed potential websites for inclusion (ranging from purely informational to highly interactive) with the CAC and our final selection was based on feedback pertaining to ease of use and clarity of presentation. 

*(2) “Functional” Definition of Health*. We presented the “functional” definition of health articulated during the adolescent interviews and the CAC concurred with the preference to encourage individual autonomy and goal setting rather than emphasize negative outcomes associated with poor health. The CAC adolescent participants were clear that the kids in the DVD needed to be “real teens” (both overweight/obese and not overweight/obese teens) dealing with challenges of weight. This strategy was incorporated into the DVD by having CAC participants and friends appear in the DVD and briefly describe their struggles as well as the internal sources of motivation that inspired them to make changes. 

*(3) Barriers to Weight Loss in School*. The CAC recognized the challenge to achieving weight loss in the school environment. The group advocated that the DVD contain practical nutritional information to help them make healthier choices amongst likely food offerings (e.g., pizza, burritos, bagels, etc.). Websites containing further information were also referenced in both the DVD and health care provider toolkit. 

*(4) Strategies to Achieve Weight Loss*. As described above, teens indicated that any effort to achieve weight loss had to begin with an individual identifying some type of internal motivation and, ideally, having a supportive network of friends and family to sustain the effort. While the CAC agreed with this perspective and specific strategies were included in the DVD to encourage recognition of this internal motivation, the group also advocated for the inclusion of physical activities to “jumpstart” the process. Therefore, following the input of both the interviewees and the CAC, brief instructional segments featuring dance, kickboxing, and weight lifting were added to the DVD. 

*(5) Parent Views on Changing the Home Environment*. Adolescents and parents in the CAC agreed that it was not sufficient to only focus on the school environment. The group offered a series of strategies for involving parents in a practical and appropriate way during the ACTION intervention. This included having the school health provider call the parent to provide regular updates, distribution of a short parent newsletter, and ideas for affordable and quick healthy recipes for busy families. 

*(6) DVD Content*. Toward the conclusion of the six CAC sessions in the first year of the study, the group reached consensus on three sections to feature on the DVD: (1) adolescent motivation for change, (2) strategies targeting energy balance and nutritional quality, and (3) physical aerobic dance and strength/resistance training instructional segments. The CAC reviewed each of the elements to be included in the DVD as well as offered stylistic suggestions relating to background music and video editing techniques to appeal to adolescents. 

*(III) Use of the ACTION DVD and Toolkit*. At the conclusion of the six CAC sessions, coordination and filming of the DVD were led by the video producer. Many of the CAC members—teens and parents—participated in this process and appear in the final DVD either sharing their own personal stories or presenting the nutrition or physical activity strategies. The ACTION Toolkit contained a parent newsletter and adolescent session tools (e.g., goal setting, internet resources, activity/food journal, etc.). 

*(IV) ACTION Outcomes and Satisfaction Results*. While the focus of this paper is to report on the process of developing the ACTION intervention materials, we are also able to offer a brief overview of process evaluation findings related to participant satisfaction with these components as well as the primary outcome measure. 

Health outcome measures for the ACTION study showed that students receiving the intervention had greater prepostimprovements in BMI percentile (*P* = 0.04) and waist circumference (*P* = 0.04) as compared to the standard care control group. BMI median percentile decreased 0.3% in the intervention group while the standard care control group's BMI median percentile increased by 0.2%. Mean waist circumference in the intervention group remained unchanged and there was a 1.7 cm increase in the standard care group. While these outcome measures are important, we also conducted process evaluation aimed at elucidating factors that may help us to better understand how the use of this adaptive CBPR approach relates to these results. 

Students reported high levels of satisfaction with the materials used in the intervention. On a scale of 0 (not at all useful) to 5 (very useful), students were asked to rate the usefulness of the DVD (*N* = 26; mean score 3.1), the clinician toolkit handouts (*N* = 27; mean score 4.0), and their overall satisfaction with the intervention (*N* = 28; mean score 4.4). Similarly, parents expressed satisfaction with their involvement in the ACTION project. Parents favorably rated the usefulness of the parent publication (*N* = 18; mean score 3.6), the usefulness of the clinician toolkit handouts (*N* = 23; mean score 3.7), and their overall satisfaction with the intervention (*N* = 25; mean score 4.4). Lastly, process evaluation findings reveal high rates of retention among participants in both the intervention group (90%) and standard care control group (79%). 

## 4. Discussion

We used formative assessment research guided by an adaptive CBPR approach to create an SBHC obesity intervention program. The student and parent interviews generated initial thematic findings that were used to guide discussions in the subsequent CAC sessions toward the development of the intervention DVD and clinician toolkit. This approach enabled us to accomplish the following goals in the first year of this study: (1) gain a better understanding of the school setting with regard to barriers to physical activity and healthful food choices, (2) create strategies/materials to address these barriers, and (3) develop culturally appropriate intervention materials and approaches based on input from study participants. This intervention led to promising health outcomes and satisfaction with materials. 

We identified several elements that may be useful in subsequent efforts to develop and/or further refine obesity intervention materials in school-based settings. Adolescents emphasized the importance of media, particularly use of the internet, as a primary source for most informational needs. We also observed that these culturally diverse (Hispanic, American Indian, and African American) adolescents expressed mostly similar views about the types of barriers and proposed solutions to addressing overweight/obesity both at home and in the school. There was strong consensus regarding a “functional” definition of health that focused on identifying internal sources of motivation to achieve goals and engagement in activities as opposed to one based on more standard biomedical measures of health (e.g., blood pressure or cholesterol levels). Partnering with these teens and adolescents was essential in order to ensure not only the appropriate content in the ACTION intervention but also the stylistic elements that maximize appeal and likelihood of use. We believe that the high levels of satisfaction reported by ACTION participants, as well as the promising results in the primary outcome measures, are attributable to this adaptive CBPR process. 

### 4.1. Implications for School-Based Obesity Research

Over the past several years, increasing attention has been directed toward schools settings as promising venues for obesity prevention and treatment interventions. The recently released Institute of Medicine report identified schools as catalysts in our efforts to accelerate progress in obesity prevention [9]. A growing body of literature supports the value of directing programmatic and research resources to school-based interventions. Prior research has demonstrated the efficacy of a quality improvement initiative aimed at enhancing SBHC provider implementation of obesity treatment guidelines [29]. Another recently published study reports findings from an adolescent obesity screening program conducted through a school-based health center [30]. In this study, following a medical evaluation, adolescents determined to be outside “healthy ranges” were referred to healthcare services. While these types of quality improvement and screening and referral projects are consistent with the need to engage schools in obesity prevention efforts, there is still a gap in the development and implementation of obesity interventional research through SBHCs. We believe the ACTION study is the first to actually create and test such an intervention through a partnership with key stakeholders located in the school community.

Conducting research in partnership with the SBHCs, the school administration, the students, and their families poses a set of logistical and study design challenges. The ACTION study involved each of these key stakeholders in different ways and at different stages to both maintain relationships that preceded this project and establish new ones to sustain our efforts going forward. The adaptive CBPR design employed in the ACTION study may offer useful guidance to other researchers conducting research in school settings. While much CBPR is oppositely structured—forming the community advisory group first—in this two-year study it was imperative that the formative research and production of the intervention materials (DVD and clinician toolkit) are completed by the start of the intervention, coinciding with the school calendar. Our goal was to incorporate the core principles of CBPR while operating in a compressed timeframe imposed by the research funding. As evidenced from both the process evaluation findings and health outcome measures, we believe that these adaptations were essential to create an authentic partnership while adhering to a timeline that may not have been feasible using a more traditional CBPR approach. While use of CBPR appears to enhance the effectiveness of interventions, there exists a lack of clarity regarding how different types of partnership configurations and processes contribute to these outcomes [26].

### 4.2. Limitations

We have identified a few limitations to this study. The first relates to the limited generalizability of these findings to other populations and school-based settings. Our purpose in this paper, however, is to demonstrate the applicability of an adaptive CBPR process through which researchers can identify locally and contextually relevant factors in their school-based obesity interventions. The second limitation involves the relatively small sample size of interviews in the formative assessment phase. The iterative process of data collection and analysis led to data saturation at an early stage and the consistency of thematic responses enhanced the confidence of findings from ethnoracially diverse students and parents. 

## 5. Conclusion

CBPR partnerships with school-based communities represent an important opportunity for researchers seeking to address a broad spectrum of adolescent health problems. There is an urgent need to find innovative, cost-effective, and sustainable strategies to reach underserved communities. We found that conducting formative assessment using an adaptive CBPR process with a range of school-based stakeholders was an effective way to develop culturally appropriate and tailored intervention materials and approaches in these multiethnic school settings. We demonstrated that researchers can adapt their study designs in ways that best maximize limited resources and time constraints without compromising the core principles of CBPR. More encouraging were promising results from this intervention in this compressed time period. 

## Figures and Tables

**Table 1:** Research activities associated with CBPR Principles.

CBPR principle	Research Activities
Acknowledging the school community as a unit of identity	Interact with full range of school representatives including administrators, SBHC personnel, students, and teachers
Building on strengths and resources within the school community	Intervention developed from prior work in target schools; use available resources (SBHC)
Facilitating collaborative partnerships in all phases of research	Meet regularly with all key stakeholders in participatory design
Fostering colearning and capacity building among all partners	Iterative process to review emergent themes and reach consensus on intervention strategies
Focusing on local relevance of the public health problem of obesity	ACTION study focus derived from key school system stakeholders
Involving a cyclical and iterative process	Each stage of research codeveloped, reviewed and, approved by partners
Involving a long-term process and commitment to sustainability	Members of the university team (UNM SBHC staff and PI) have over a decade of involvement in the school settings

**Table 2:** Demographic characteristics of student and parent interviewees.

	Students (*N* = 7)	Parents (*N* = 8)
Participating schools		
Intervention	2	3
Control	5	5
Sex		
Female	4	6
Age (mean years)	16	45
Ethnicity		
Hispanic	3	3
African American	2	2
American Indian	1	1
Non-Hispanic white	1	1
Other	0	1
Employment status		
Full time	0	4
Part time	3	2
Not employed	4	2

**Table 3:** Key informant themes and resulting intervention strategies.

Theme	Resulting Strategies
Media use	Include healthy weight and physical activity web sites in DVD and provider toolkit

“Functional” definition of health	(i) Include “real kids” (overweight/obese and non-overweight/obese) in DVD (ii) Documentary-style interviews with adolescents discussing their reasons for eating healthier and being physically active in DVD

Barriers to weight loss in schools	(i) Add practical nutritional information in DVD and toolkit that match food offerings in schools to facilitate better nutritional choices (ii) Food displays in clinic to facilitate discussions on how to choose healthier options

Strategies to achieve weight loss	(i) Promote emphasis of weight loss as consistent with personal, internal sources of motivation (ii) Incorporate brief instructional segments in DVD consistent with adolescent interests (e.g., dance, kickboxing, strength/resistance training)

Parent views on changing home environment	(i) Create mechanism for communication between health care provider and parents to provide regular updates and reinforce ACTION themes (ii) Distribute parent newsletter and healthful recipes

Input on DVD content	Ensure that DVD featured three sections: (1) adolescent motivation for change; (2) strategies targeting energy balance and nutritional quality; and (3) physical aerobic dance and strength/resistance training segments
